# Impaired corrective responses to postural perturbations of the arm in individuals with subacute stroke

**DOI:** 10.1186/1743-0003-12-7

**Published:** 2015-01-20

**Authors:** Teige C Bourke, Angela M Coderre, Stephen D Bagg, Sean P Dukelow, Kathleen E Norman, Stephen H Scott

**Affiliations:** Centre for Neuroscience Studies, Queen’s University, Kingston, ON Canada; Department of Physical Medicine and Rehabilitation, Queen’s University, Kingston, ON Canada; Clinical Neurosciences, University of Calgary, Calgary, AB Canada; School of Rehabilitation Therapy, Queen’s University, Kingston, ON Canada; Department of Biomedical and Molecular Sciences, Queen’s University, Kingston, ON Canada

**Keywords:** Stroke, Proprioception, Assessment, Perturbation, Upper limb, Robotics

## Abstract

**Background:**

Stroke is known to alter muscle stretch responses following a perturbation, but little is known about the behavioural consequences of these altered feedback responses. Characterizing impairments in people with stroke in their interactions with the external environment may lead to better long term outcomes. This information can inform therapists about rehabilitation targets and help subjects with stroke avoid injury when moving in the world.

**Methods:**

In this study, we developed a postural perturbation task to quantity upper limb function of subjects with subacute stroke (n = 38) and non-disabled controls (n = 74) to make rapid corrective responses with the arm. Subjects were instructed to maintain their hand at a target before and after a mechanical load was applied to the limb. Visual feedback of the hand was removed for half of the trials at perturbation onset. A number of parameters quantified subject performance, and impairment in performance was defined as outside the 95th percentile performance of control subjects.

**Results:**

Individual subjects with stroke showed increased postural instability (44%), delayed motor responses (79%), delayed returns towards the spatial target (79%), and greater endpoint errors (74%). Several subjects also showed impairments in the temporal coordination of the elbow and shoulder joints when responding to the perturbation (47%). Interestingly, impairments in task parameters were often found for both arms of individual subjects with stroke (up to 58% for return time). Visual feedback did not improve performance on task parameters except for decreasing endpoint error for all subjects. Significant correlations between task performance and clinical measures were dependent on the arm assessed.

**Conclusions:**

This study used a simple postural perturbation task to highlight that subjects with stroke commonly have difficulties responding to mechanical disturbances that may have important implications for their ability to perform daily activities.

## Background

When holding a drink in a crowded room and someone bumps your arm, you must rapidly respond to keep the drink from spilling. Recent studies highlight that the motor system is capable of generating intelligent corrective responses to unexpected forces applied to the body [1, 2]. For example, perturbation responses have been shown to account for subject intent [3–7], urgency to respond [8], properties of the limb [9] and properties of the goal and environment [10, 11]. These task-related responses can be observed in the long-latency time period, ~50 ms after perturbation onset, implying these responses are generated through a transcortical feedback pathway [12–14]. Historically, the transcortical feedback pathway is considered to involve primary motor and somatosensory cortices [15–17], but many other cortical and subcortical areas may contribute to these corrective responses [2].

Stroke can damage cortical and subcortical regions of the brain or spinal cord, leading to a wide range of sensory and/or motor impairments [18]. This damage often leads to patients having slow, jerky and uncoordinated movements post-stroke [19]. The impact of stroke on corrective responses in the upper limb has principally been explored by quantifying long latency stretch responses in muscles. In stroke, long-latency responses have been found to be delayed and/or absent in wrist muscles [20] and the biceps brachii [21]. Subjects with stroke do not modulate their long latency responses when their arms counter stiff versus compliant environments [10] and also display inappropriate coupling between different muscle groups [22].

Only a few of the aforementioned studies that quantified changes in long-latency stretch responses in the upper limb also examined behavioural responses to perturbations [14, 19]. Many perturbation studies were designed to assess spasticity and instructed subjects to relax and not intervene or react to the perturbation [14, 19, 23–25]. Other studies have instructed subjects to actively assist [26] or actively resist [27] the perturbation. In these paradigms the applied perturbation directly controlled limb motion so that the subject could not actively achieve the behavioural goal. Therefore, it is difficult to interpret the subjects’ behavioural impairments without an attainable goal [2].

Previous studies also highlight potential deficits in the ipsilesional arm post-stroke. For example, subjects with stroke have shown bilateral impairments in modulating long latency responses between stiff and compliant environments [10]. Marsden and colleagues also found that 5 out of 12 subjects with stroke had reduced long latency responses in the both arms [15]. Again, it is not clear how altered long-latency responses in the ispilesional limb are linked to behavioural impairments for these individuals.

Finally, vision plays an important role in voluntary motor control and could provide an important alternate source for correcting limb disturbances when somatosensory feedback is impaired. Bonan and colleagues found that visual feedback was critical for maintaining whole-body posture [28]. Piovesan and colleagues found arm stiffness was reduced during reaching when post-stroke subjects used visual feedback [29]. However, little is known on the relative contribution of visual and somatosensory feedback to counter limb perturbations [30]. Visual feedback is slower than limb somatosensory feedback. Thus it is predicted that impairments in somatosensory feedback can be compensated for by visual feedback except for a slight delay in the corrective response.

Our goal was to create a behavioural task to quantify the ability of subjects with stroke to actively correct for unexpected disturbances of the arm during a goal-directed motor action. Subjects had to maintain their hand at a spatial goal and a constant load was applied to the limb so that subjects must respond to the disturbance to achieve the behavioural goal [2]. Corrective responses were assessed in both the contra- and ipsilesional arms. As well, we examined corrective responses with and without vision to quantify whether impairments in the use of somatosensory feedback could be compensated for with vision. Subjects with stroke were often slower than controls in decelerating the arm in response to the imposed load, took longer to return to the goal or undershot the target. Endpoint error was the only parameter that showed improvement when visual feedback was provided to subjects. About half of subjects with stroke who showed task impairments with their more affected arm also showed impairments with their other arm. Thus, this robotic postural perturbation task quantifies post-stroke impairments in the use of limb afferent feedback to generate motor corrections.

## Methods

### Subject information

Participants with stroke were recruited from three inpatient stroke rehabilitation programs: Providence Care (St. Mary’s of the Lake Hospital, Kingston, ON), Dr. Vernon Fanning Centre (Calgary, AB) and Foothills Hospital (Calgary, AB). Subjects potentially eligible to participate were assessed 2–50 days post-stroke usually within 2 weeks of being admitted to rehabilitation programs at each centre. Prospective subjects were excluded if they had bilateral lesions, previously diagnosed strokes, other neurologic diagnoses (e.g. Parkinson’s disease), ongoing upper extremity musculoskeletal injuries, or an acute medical illness. Non-disabled control subjects were recruited from the local Kingston, ON community. All subjects were able to understand the instructions required to complete the task, were able to see the visual target, and provided informed consent. This study was approved by the Queen’s University Health Sciences and Affiliated Teaching Hospitals Research Ethics Board (#ANAT-042-05), and the University of Calgary’s Conjoint Health Research Ethics Board (#22123).

### Clinical assessments

Subjects with stroke were evaluated using a number of standardized clinical assessments. Upper limb physical impairments were measured using the Chedoke-McMaster Stroke Assessment (CMSA) impairment inventory for the arm [31]. In this scale, impairment is measured on a seven-point scale based on Brunnstrom’s stages of motor recovery [32]. Subjects received a minimum score of one for flaccid paralysis and maximum score of seven for normal voluntary movement control. Subject strength was also measured for elbow and shoulder flexion and extension using the muscle power assessment [33]. Each muscle group (shoulder and elbow) was scored from no visible or palpable contraction (0) to normal volitional isometric strength (5), for a total composite score of 20 for upper limb strength. Functional abilities were measured by the Functional Independence Measure (FIM) [34]. The FIM scores the amount of assistance (1 = total assistance, 7 = total independence) required to achieve different activities of daily living, and can be divided into cognitive and motor subsections. The conventional subtests of the Behavioural Inattention Test (BIT) were used to screen for deficits of attention/neglect with a score of less than 130/146 indicative of visual neglect [35]. Subjects were broadly categorized as “Left-Affected” (LA) or “Right-Affected” (RA) depending on the clinically most affected side of the body. All subjects took the Modified Edinburgh Handedness test to determine handedness [36].

### Experimental setup

The experimental assessment was implemented using a bilateral KINARM exoskeleton device (BKIN Technologies Ltd, Kingston, ON, Canada). Subjects sat in a modified wheelchair base and their forearms and upper arms were fitted snugly to plastic arm troughs that were attached to an adjustable four bar linkage [37, 38]. Linkage lengths were adjusted to the dimensions of the subject’s arms, permitting free movement of the elbow and shoulder joints in the horizontal plane. The exoskeleton supported the weight of the subject’s arms against gravity and was used to apply mechanical loads to the elbow or shoulder joint. Subjects were wheeled into an augmented reality system that displayed virtual targets in the same plane as arm motion. Direct vision of the subject’s arms was occluded. Visual feedback, by means of a white dot at the location of the subject’s index fingertip position, was provided.

Position and velocity of the robot was recorded at a sampling rate of 1000 Hz. Subject joint angles and velocities, and hand position, speed, and acceleration were calculated from these values. Hand and joint-based signals were analyzed using MATLAB (Mathworks Inc., Massachusetts). Signals were filtered using a sixth-order double-pass Butterworth low pass filter with a cutoff frequency of 10 Hz.

### Experimental task

Subjects were assessed in an upper limb postural perturbation task. In each trial, subjects were required to maintain their hand at a virtual target. After a random delay of 1750 to 2250 ms, subjects received a flexor or extensor step torque to the elbow (+/−0.5 Nm) or shoulder (+/−1 Nm). Subjects were instructed to return their hand to the target as soon as they felt the robot ‘bump’ them. The perturbation torque remained constant during the duration of the trial (3 seconds). A step torque was chosen to ensure subjects could not simply co-contract their muscles to respond to the perturbation. As well, the sustained torque allowed for the full unfolding of long-latency and voluntary motor responses to the perturbation which would have otherwise been quenched within 30 ms of perturbation offset [2, 39]. This meant subjects needed to actively increase muscle activity to oppose the load to bring the hand back to the target. The task was divided into 9 blocks of 8 trials each. Each block contained two trials of each of the four perturbation conditions (flexion and extension of the shoulder and elbow). In the first block (not used in analyses), subjects were able to practice with visual feedback of the fingertip position. Subsequently, visual feedback of fingertip position was removed at perturbation onset in half of the trials in each block. The order of trials was randomized within each block. Subjects completed the task with one arm (chosen at random) before completing it with the other arm.

A subset of subjects (n = 11, subjects with stroke; n = 6 controls) completed inter-rater reliability testing of the postural perturbation task. After their first assessment was completed, the subject was taken out of the chair and all adjustments to the KINARM robot were modified (e.g. seat height, arm length, calibration of video display). At this time, a second operator, not present for the initial assessment, set up the subject in the device and reassessed the same subject. The second assessment of the subject usually occurred immediately following the first assessment and at most 7 days from the original assessment. This second operator had no knowledge of the previous subject specific adjustments.

### Task performance measures

**Figure 1:**
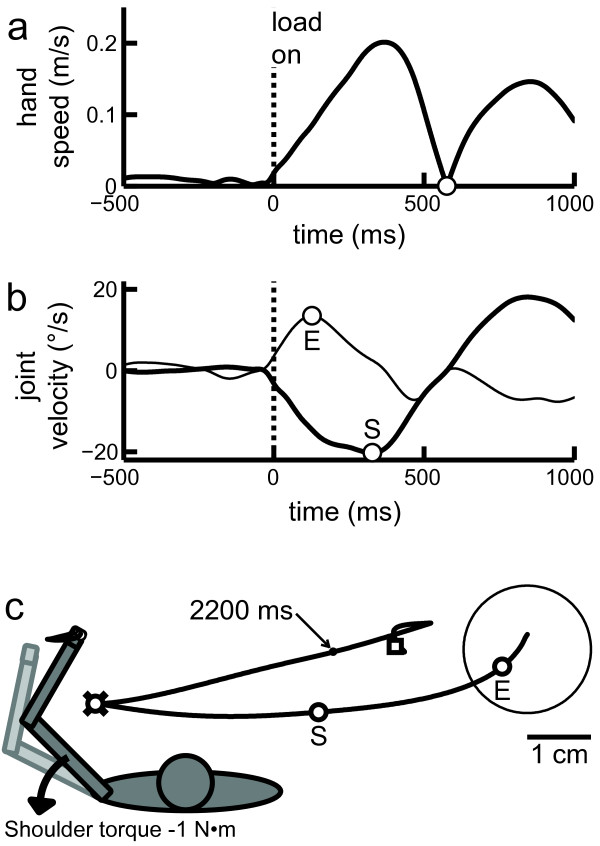
**Exemplar trial where the left arm is responding to being perturbed into shoulder extension. (a)** Hand speed over time. Hand speed minimum representing the end of the hand’s initial deceleration is shown by the circle marker. **(b)** Joint velocity of the loaded joint (shoulder, thick line) and the non-loaded joint (elbow, thin line) over time. Joint flexion is positive and joint extension is negative. The initial velocity extrema of the elbow and shoulder joint are marked by the circle markers, labelled ‘E’ and ‘S’ respectively. The offset is the time between these points. **(c)** Left: Schematic of arm orientation and perturbation direction for the trial presented in this figure. Right: Participant’s hand path after perturbation onset. The visual target and the hand path are shown in black. The initial velocity extrema of the elbow and shoulder joint are marked by the circle markers, labelled ‘E’ and ‘S’ respectively. The X marker indicates the maximum displacement of the hand (which in this case corresponded to the deceleration time- circle marker). Return time occurred when subject returned within 1 cm of their endpoint location (dot marker indicated to occur at 2200 ms). Endpoint (the position at the end of the trial) is indicated by square marker and endpoint error is the distance between this point and the centre of the visual target.

*Posture speed*- The 95th percentile of the median hand speed for the 500 ms before perturbation onset (Figure 1a). Increased postural speed indicated increased difficulty maintaining the fingertip within the visual target.

*Deceleration time*- The time it took subjects to reach the first minimum hand speed after perturbation onset (Figure 1a). Deceleration time quantified how soon subjects responded to the perturbation and opposed the imposed load enough to slow their arm. The acceleration time (time to first hand speed maxima) could also have been used to quantify the time to respond to the perturbation, but was highly correlated to deceleration time (r = 0.90, p << 10^−27^) and less sensitive to stroke-related impairments.

*Return time*- The time required for subjects to return to within 1 cm of their position at the end of the trial (Figure 1c). This indicated how quickly subjects took to complete their corrective response within the 3 seconds time limit.

*Endpoint error*- The distance from the target centre at the end of the trial (Figure 1c). Endpoint error quantified how accurately subjects could use feedback (proprioceptive, or proprioceptive and visual) to position their hand back to the target within the 3 second time limit.

*Maximum displacement*- The maximum distance the subject’s fingertip was pushed out of the target by the perturbation (Figure 1c). This measures how effectively subjects were able to resist the imposed load.

*Joint velocity offset*- The time difference between the first velocity extrema of the directly and indirectly perturbed joints. For instance, in a shoulder flexion perturbation trial, the shoulder (directly perturbed joint) may take longer to respond than the elbow (indirectly perturbed joint) (Figure 1b). Joint velocity offset was used as an indicator of the timing of multi-joint coordination while responding to the perturbation.

### Statistical analysis

The mean performance of control subjects was used to create a normative reference range for each parameter [40]. This range was used to characterize each individual stroke subject as impaired on a given parameter if they had mean performance greater than 95% of control subjects. From the distribution of mean control performance, linear regressions were used to determine if there was any effect of age or body weight. Regression residuals were tested for normality (Lilliefors test, p < 0.05). If residuals were not normally distributed, logarithmic, square root, inverse or exponential transforms of the original data were used to attempt to obtain a linear regression with normally distributed residuals. The linear regression was then transformed back into native values and used to create a normative model. A two-tailed Kolmogrov-Smirnov test (KS test) was used to determine if there was any effect of hand dominance or sex.

If the residual distribution could not be transformed to a normal distribution, a Wilcoxon Rank Sum (p < 0.05) of the original data was used to determine if these factors had any significant effect. Control data were then binned into three different ranges of age or body weight (depending on the effect) and separate percentiles for each bin created the normative model. A rank sum test was then used to determine if there was any effect of hand dominance or sex.

The 95th percentile of control performance was used to characterize an individual stroke subject as impaired in a given parameter. If performance of a control was outside the 99.9th percentile for a single parameter, the subject’s data were excluded for all parameters. If a parameter could not be transformed to have normally distributed control performance, the threshold for exclusion was 2x the 95th percentile. This exclusion step identified control subjects who were outliers in any of the 6 task parameters. After each exclusion of an outlier, the threshold percentiles for exclusion were re-calculated and any new outliers were identified and excluded. This process continued until no further outliers could be identified. In total, 13 control subjects were excluded from further analysis.

## Results

### Subject demographics and clinical measures

Demographic and clinical information about the subjects are provided in Table 1. Thirty eight subjects with sub-acute stroke completed the perturbation task. All subjects had an ischemic stroke except for one subject who had a left hemisphere hemorrhagic stroke. Our group of subjects with stroke displayed a broad range of FIM scores (37–126), but a median score of 107 indicated many subjects had mild disability (108 for LA subjects and 107 for RA subjects). Five subjects with stroke had a BIT score of less than 130 indicative of neglect, and 8 subjects had visual field deficits (3 with hemianopsia, 1 quandrantanopia, 3 peripheral vision loss, and 1 smaller scotoma). Stroke subjects’ CMSA arm scores ranged from 2–7 for the affected arm and 5–7 for the unaffected arm.

**Table 1:** **Clinical and demographic information of included subjects**

Measure	Subjects with Stroke (n = 38)	Left-Affected (n = 22)	Right-Affected (n = 16)	Controls (n = 74)
Age (years)^a^	63.5 (26–87)	65.5 (26–83)	62 (41–87)	47.5 (20–87)
Weight (kg)^a^	85.2 (55–127)	85.2 (55–127)	85.4 (59–127)	75 (45.5-134)
Sex (M/F)	21/17	12/10	9/7	42/32
Handedness (L/R/A)	6/32/0	2/20/0	4/12/0	6/68/0
Days since stroke^a^	27.5 (2–50)	22.5 (2–46)	31.5 (12–50)	-
FIM^ab^	107 (37–126)	108 (37–124)	107 (66–126)	-
FIM motor score^ac^	74.5 (17–91)	75.5 (17–90)	73.5 (41–91)	-
BIT score^a^	141.5 (86–146)	141.5 (86–146)	142 (128–146)	-
Strength^a^	16 (3–20)	16.5 (3–20)	16 (4–20)	-
CMSA arm score				
Affected arm^d^	[0 8 9 5 8 4 4]	[0 4 4 2 7 3 2]	[0 4 5 3 1 1 2]	-
Unaffected arm^d^	[0 0 0 0 6 16 16]	[0 0 0 0 5 6 11]	[0 0 0 0 1 10 5]	-

Abbreviations: M/F (male/female), L/R/A (left/right/ambidextrous), FIM (Functional Independence Measure), CMSA (Chedoke-McMaster Stroke Assessment scale) Legend: ^a^median (min-max); ^b^FIM consists of 18 items scored from 1 to 7, higher scores representing greater functional independence ^c^subset of FIM with 13 items. ^d^[n1 n2 n3 n4 n5 n6 n7] corresponds to the number of subjects with CMSA arm scores of [1 2 3 4 5 6 7]. A higher score represents lower physical impairment.

Eighty-seven controls were also assessed in the postural perturbation task, of which 74 met the criteria for retention (see Methods- Statistical Analysis). Subjects were specifically selected to span a broad range of ages in order to develop age normative models (see Methods- Statistical Analysis). The control group was similar to the group of subjects with stroke in its proportions for sex (controls: 57% male, subjects with stroke: 55% male) and reported hand dominance (controls: 92% right hand dominant, subjects with stroke: 82% right hand dominant).

### Task results without visual feedback of hand position

#### Exemplar subjects

**Figure 2:**
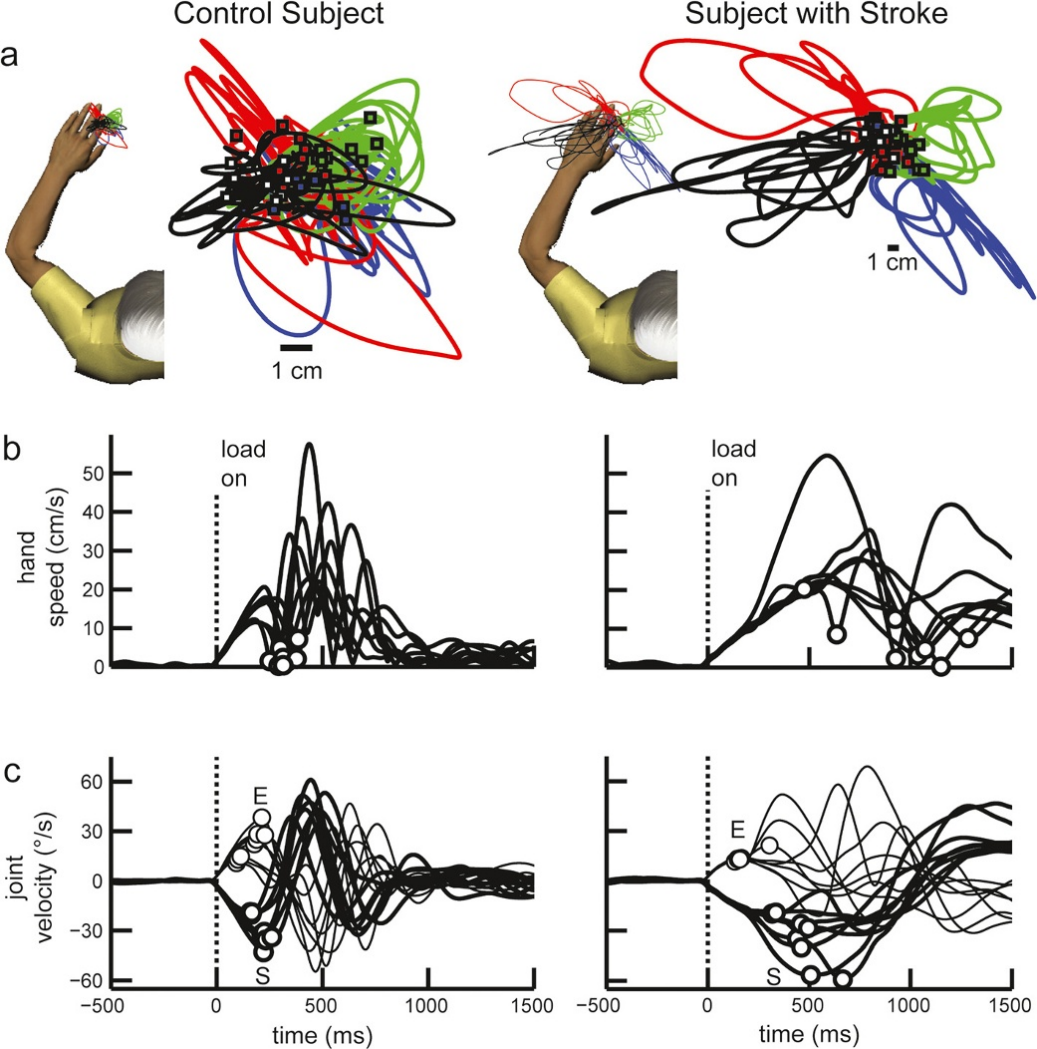
**Performance of exemplar subjects in postural perturbation task.** Left panel: Control subject. **(a)** Hand paths with left arm. Trials responding to an elbow flexion, elbow extension, shoulder flexion, and shoulder extension perturbation are shown in blue, red, green, and black, respectively. Square markers show endpoint positions. **(b)** Subject hand speed. Circles are first hand speed minima. **(c)** Subject elbow (thin line) and shoulder (thick line) velocity. First peaks are represented by circle markers, labelled ‘E’ and ‘S’ respectively. Right panel: Subject with stroke. **(a)** Hand path with left affected arm. Note that subject hand paths are on different scales between panels. **(b)** Hand speed for subject with stroke. **(c)** Joint velocity for subject with stroke.

The left panels of Figure 2 displays perturbation responses of an 83 year old female control subject who was right handed. Performance is shown for the left arm for trials where vision of the hand is removed at the start of the perturbation. Hand motion was minimal following perturbations (mean maximum displacement: 3.85 cm) and the subject’s hand was, on average, within 1.32 cm of the centre of the target at the end of the trial (mean return time: 1051 ms) (Figure 2a). Posture speed for this subject was 0.84 cm/s. Figure 2b displays hand speed when shoulder extensor loads were applied. Initial hand speed profiles display a similar first peak and first minima, highlighting that the subject generated a consistent rapid motor response to stop hand motion at ~300 ms following the shoulder perturbation. Beyond this time, speed profiles are more idiosyncratic when returning and maintaining their hand within the spatial target. Shoulder and elbow velocity also followed a consistent pattern when this shoulder extension perturbation was applied, with the elbow flexor velocity first peaking at ~100-200 ms followed by the shoulder extensor velocity peaking at ~150-250 ms (Figure 2c).The right panel of Figure 2 displays perturbation responses from the left affected arm of an 80 year old female measured 27 days post stroke. This subject was right handed, had a FIM motor subscore of 68/91, and scored 6/7 on the CMSA arm subscale with their left arm. The hand of the subject with stroke was displaced 11.90 cm (on average) outside the target (Figure 2a) by the perturbation. This subject took much longer to return (mean return time: 2380 ms) than the control subject, and often ended the trial short of the target resulting in average endpoint errors of 1.84 cm (Figure 2a). Postural hand speed was also greater for this subject at 1.30 cm/s. The first hand speed peaks and minima of this subject with stroke were later and more variable than the control subject, highlighting the difficulty in stopping her hand against the imposed load (Figure 2b). The first elbow velocity peak showed similar timing to the control subject, peaking at ~150 ms after the perturbation (Figure 2c). However, the first shoulder velocity peaks of this subject with stroke were more delayed and variable following the perturbation. This resulted in larger and more variable joint velocity offsets.

#### Control performance

For each parameter, the average performance of control subjects was used to quantify typical behavior. Only return time showed a significantly greater values for females as compared to males (KS test, *D* = 0.30, *p* < 0.01). Endpoint error significantly increased with age (linear regression, *r* = 0.25, *p* < 0.01). Path length and joint velocity offset (elbow and shoulder perturbation conditions) significantly increased with body weight (linear regression, *r* = 0.25, *r* = 0.14, *r* = 0.36, *p* < 0.01). Return time significantly increased with age for the female subgroup (linear regression, *r* = 0.43, *p* < 0.01). Return times also significantly decreased with body weight (i.e. heavier arms returned faster) for the female subgroup (linear regression, *r* = 0.37, *p* < 0.01). Hand dominance had no effect on the performance of control subjects (KS test, *D* < 0.17, *p* > 0.25) in any task parameter. Normative models were used to account for these effects and calculate the percentiles for these parameters. Subjects with stroke were identified as impaired if their performance was greater than the 95th percentile of controls.

Control subjects displayed a range of how quickly and accurately they could respond to the perturbation and return to the target without visual feedback of hand position. Control subjects maintained posture at the visual target with a mean hand speed ranging from 0.5 to 1.5 cm/s. After perturbation onset, 95% of control subjects stopped decelerating their hand in less than 400 ms and returned within 1 cm of their endpoint in less than 1.4 seconds. Even without visual feedback of hand position, 86% of control subjects returned their hand to the target (mean endpoint error less than 1 cm). After applying the age normative model, the 95th percentile of control performance ranged from 1.1 cm for young adults to 1.5 cm for older adults. Control subjects were usually displaced 1 to 4 cm from the target center and had a mean path length of 5–15 cm.

#### Subjects with stroke: performance with affected limb

**Figure 3:**
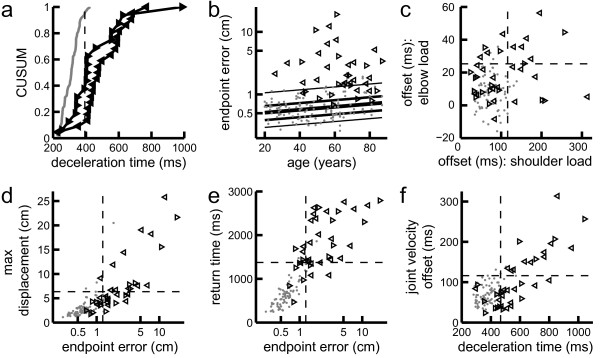
**Mean subject performance in task parameters. (a)** Cumulative sum distribution for mean deceleration time. Performance by control subjects shown in grey. Performance of the affected arm of subjects with stroke is shown by the leftward and rightward pointing triangles representing left-affected and right-affected subjects, respectively. Dotted line represents 95th percentile for controls. **(b)** Subject age versus mean endpoint error. Percentile bands of age normative model are the lines (from bottom to top) 5th, 25th, 50th, 75th, and 95th. Note the logarithmic y-axis. **(c)** Mean joint velocity offset during shoulder versus elbow perturbation trials. Note axes are not equally scaled. **(d)** Mean endpoint error versus maximum displacement. Note axes are not equally scaled and the x-axis is logarithmic. **(e)** Mean endpoint error versus return time. Note the logarithmic x-axis. **(f)** Mean deceleration time versus joint velocity offset for shoulder load trials.

Many individual subjects with stroke showed greater parameter values with their affected arm than 95% of controls and were identified as impaired (Table 2). Interestingly, left affected (LA) subjects were impaired more frequently than right affected (RA) subjects for all parameters. For instance, 91% of left affected subjects and 63% of right affected subjects had impaired deceleration times (Figure 3a). Similarly, as compared to the age normative model, 86% of LA subjects and 56% of RA subjects had impaired endpoint errors (Figure 3b).

**Table 2:** **Task performance**

Parameter		LA impaired (%)		RA impaired (%)		Interrater r (p)
		A arm	U arm	A arm	U arm	
Posture speed		50	41	38	13	.74 (<10^−4^)
Deceleration time		91	50	63	44	.68 (<10^−4^)
Return time		91	77	63	44	.91 (<10^−11^)
Endpoint error		86	68	56	38	.91 (<10^−11^)
Maximum displacement		55	50	31	19	.97 (<10^−14^)
Joint velocity offset	elbow load	36	32	19	19	.81 (<10^−7^)
	shoulder load	45	41	25	19	.74 (<10^−6^)

*Abbreviations: LA* (left affected subjects), *RA* (right affected subjects), impaired (subjects with stroke whose performance exceeded 95% of controls on a particular task parameter), A arm (affected arm), U arm (unaffected arm).

Although elbow and shoulder loads created significantly different responses in each task parameter for controls (KS test, *D* > .39, *p* < 10^−4^) the intra-subject values were highly correlated (controls: *p* < 10^−12^, *r* > =0.71, stroke: *p* < 10^−7^, *r* > =0.74). Joint velocity offset was the one parameter that was an exception to this finding (Figure 3c). In response to a shoulder perturbation, the shoulder always took longer to reach its first velocity extrema than the elbow. In response to an elbow perturbation, the elbow reached its first velocity extrema at about the same time as the shoulder (extrema were within 10 ms of each other for 57% of controls and 39% of subjects with stroke). Reflective of these differences, joint velocity offset was poorly correlated between elbow and shoulder perturbation trials for both controls (*r* = .08, *p* = 0.49) and subjects with stroke (*r* = .23, *p* = 0.17). Further, more subjects (32%) were impaired only in response to an elbow or a shoulder load than were impaired in response to both loads (18%). Percentages were similar when the body weight normative model was applied.

Correlation values between deceleration time and joint velocity offset values were calculated for both stroke and control subgroups to determine if increased joint velocity offset was associated with delayed deceleration times. Deceleration time was moderately correlated to joint velocity offset in control subjects (Pearson correlation; elbow perturbation trials: *r* = 0.55, *p* < 10^−6^; shoulder perturbation trials: *r* = 0.54, *p* < 10^−6^) and highly correlated in subjects with stroke (Pearson correlation; elbow perturbation trials: *r* = 0.74, *p* < 10^-7^; shoulder perturbation trials: *r* = 0.79, *p* < 10^−8^) (Figure 3f). Therefore, the delayed response of the perturbed joint relative to the unperturbed joint may contribute to delays seen in many subjects with stroke in deceleration time.

Similarly, correlations between maximum displacement and endpoint error were calculated for both stroke and control subgroups to determine if increased maximum displacement was associated with greater endpoint errors. Maximum displacement showed moderate correlation with endpoint error for both controls (Pearson correlation, *r* = .74, *p* < 10^−13^) and subjects with stroke (Pearson correlation, *r* = .83, *p* < 10^−9^) (Figure 3d). Furthermore, all but one of the subjects identified as impaired in maximum displacement also had impaired endpoint errors. Return times for controls and subjects with stroke were also both correlated with their endpoint errors (Pearson correlation, *r* = 0.87, *p* < 10^−22^ and *r* = 0.53, *p* < 10^−3^, respectively). Despite a large number of subjects with stroke being impaired in both parameters, 4 and 5 subjects were identified as being impaired in only return time or endpoint error, respectively (Figure 3e). Therefore, impaired endpoint errors are likely often due to undershooting the target by the end of the trial.

### Impairments observed bilaterally

**Figure 4:**
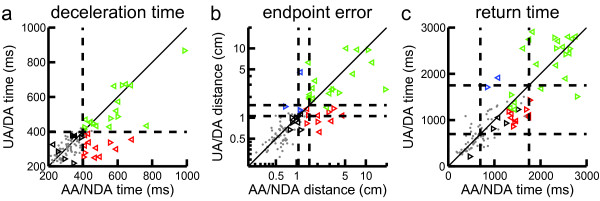
**Comparison of mean subject performance in task parameters between arms. (a)** Deceleration time. Control subject performance is shown by the grey dots with the x-value for the non-dominant arm (NDA) and the y-value for the dominant arm (DA). Performance of subjects with stroke is shown by the leftward and rightward facing triangles representing left-affected and right-affected subjects (respectively) with x-values for the affected arm (AA) and the y-values for the unaffected arm (UA). Subjects with stroke whose performance is above or below the 95th of control performance (dashed lines) for both arms are represented by green or black triangles, respectively. Subjects with stroke who exceeded the 95th of control performance for with their affected or unaffected arms only are represented by red or blue triangles, respectively. **(b)** Mean endpoint error for both arms. Dashed lines represent the 95th percentile of control performance for the minimum and maximum age according to the age normative model. **(c)** Mean return time. Dashed lines represent the minimum and maximum possible 95th percentile of control performance as according to the sex specific normative models.

Subjects with stroke had task-related impairments with their clinically defined unaffected arm. In fact, 63% of subjects showed longer return times and 55% showed greater endpoint errors with their ‘unaffected’ arm than 95% of controls. On other parameters 26-47% of subjects with stroke exhibited task related impairments with their ‘unaffected’ arm.By comparing task performance across arms, we could observe the relative magnitude of impairment in the affected versus unaffected arm (Figure 4). Although a few subjects displayed impairments in only the unaffected arm, these values were very close to 95% of controls and relate to the tradeoff of sensitivity and specificity in our impairment assignment. Most of the subjects with stroke who were bilaterally impaired in deceleration time, endpoint error, and return time can be seen to have qualitatively similar parameter values when comparing the affected and unaffected arm (Figure 4).

The presence of task impairments in the ‘unaffected’ arm could reflect our inclusion of subjects with brainstem strokes and subjects with CMSA scores <7 on the ‘unaffected’ limb (n = 23) indicating difficulties to perform certain tasks with this limb. However, we found that 40-73% of subjects with stroke who had a CMSA of 7 for their ‘unaffected’ limb and did not have a brainstem stroke (n = 15) still displayed impairments in their ‘unaffected’ arm (9 impaired on deceleration time, 6 impaired on endpoint error, and 11 impaired on return time). Qualitatively, the subjects who showed bilateral task impairments displayed a similar degree of symmetrical task performance between arms, regardless of the subgroup to which they belonged.

### Interrater reliability of robotic task performance

Interrater reliability of parameters was evaluated using an intraclass correlation coefficient. Reliability coefficients ranged from 0.68 to 0.97, indicating good to excellent reliability (see Table 2). Lower reliability values were generally associated with parameters that had a relatively small range of values across the control and stroke populations.

### Effects of visual feedback on robotic task performance

**Figure 5:**
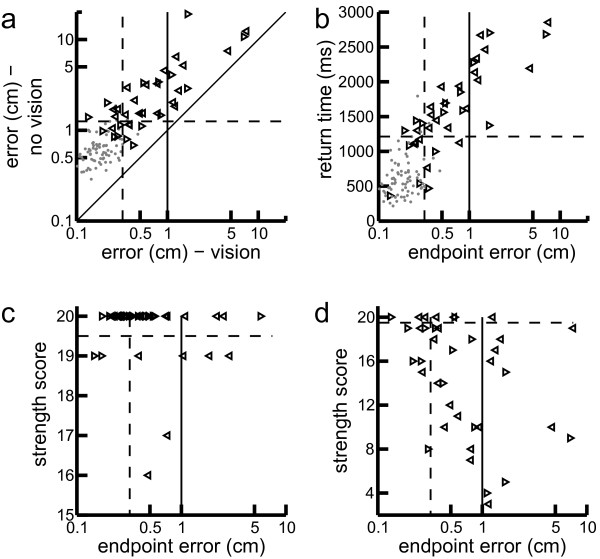
**Differences in endpoint error depending on visual feedback condition. (a)** Mean endpoint error for trials with hand feedback (vision) compared to trials with no hand feedback (no vision). Performance by control subjects is shown by the grey dots. Stroke performance of the affected arm is shown by the leftward and rightward pointing triangles representing left-affected and right-affected subjects respectively. Dotted line shows 95th percentile of control performance and solid vertical line shows target radius. **(b)** Mean endpoint error for trials with hand feedback (vision) compared to mean return time for the affected arm. **(c)** Mean endpoint error for trials with hand feedback (vision) compared to strength scores in the clinically defined affected arm. **(d)** Same as **(c)** but for ‘unaffected’ arm.

All subjects showed smaller endpoint errors when visual feedback was provided (Figure 5a), and 11 subjects with stroke ended their movements outside of the target with visual feedback. However, 17 of those subjects that returned to the target did not stabilize as close to the centre of the target as 95% of controls. Overall, 77% of left-affected and 63% of right-affected subjects with stroke had endpoint error impairments with visual feedback. All 5 subjects with BIT < 130 and all but two of subjects with visual field deficits had endpoint errors with visual feedback. Once excluded, 67% of left-affected and 64% of right-affected subjects with stroke had endpoint error impairments with visual feedback.The impairments with visual feedback in these subjects could not be explained by strength impairments. Although 10/11 subjects who stabilized outside of the target with their affected arm had some strength impairment, 21/27 of the subjects with stroke who stabilized inside the target also had strength impairments (Figure 5c). Also, all subjects who stabilized outside the target with their unaffected arm had strength scores of 19 or 20/20 (Figure 5d).

Greater endpoint errors with visual feedback were correlated with delayed return time for controls (*r* = 0.32, *p* = 0.005), and subjects with stroke (affected arm: *r* = 0.63, *p* < 10^−4^; unaffected arm: *r* = 0.70, *p* < 10^−5^) (Figure 5b). There was no effect of visual feedback manipulations for other task parameters for either controls (*D* < .11, *p* > 0.75) or subjects with stroke (*D* < .19, *p* > 0.49) according to paired KS tests.

### Relationship between standard clinical assessment measures and task performance

We expected clinical assessment values to correlate with task performance values for the affected arm and potentially for the unaffected arm. Surprisingly, task performance values from subjects with stroke were not correlated to their scores on clinical scales with their affected arm, but were sometimes correlated to those with their ‘unaffected’ arm. Specifically, posture speed of the unaffected arm correlated with its CMSA arm subscore (*r* = −0.43, *p* < 0.01), its strength (*r* = −0.55, *p* < 10^−3^) and the subject’s FIM motor subscore (*r* = −0.44, *p* < 0.01). Also, endpoint error and joint velocity offset for shoulder perturbation trials (with the unaffected arm) significantly correlated with the subject’s BIT score (*r* = −0.38, *p* = 0.02 and *r* = −0.36, *p* = 0.03, respectively). Negative correlations indicated, as expected, that greater parameter abnormality was associated with greater clinical impairment or disability.

## Discussion

Stroke can damage a wide range of brain areas, leading to disruption of many different pathways including those related to somatosensory feedback for motor function. The present study quantified the ability of subjects with subacute stroke to counter mechanical perturbations during postural control of the arm. We found that the majority of subjects with stroke were impaired in a number of parameters as compared to control subjects. Without vision, subjects with stroke commonly displayed slower responses to oppose the load, longer return times or failed to return to the target, and disrupted coordination of the shoulder and elbow. About half of the subjects with stroke that were impaired with their ‘affected’ arm were also impaired with their ‘unaffected’ arm. Visual feedback did not improve corrective responses except for endpoint error, although impairments in endpoint error could persist even with visual feedback. Ipsilateral impairments were correlated with clinical measures, indicating bilateral impairments may be associated with greater overall impairments and disability.

### Impairments in multi-joint coordination during corrective responses

The coordination of the elbow and shoulder joints is an important component of voluntary control, as successful motor actions must account for the fact that torque produced at one joint results in an interaction torque at other joints [41]. Healthy subjects can deal with this biomechanical property of a multi-segmented arm generating consistent temporal patterns of joint kinematics during voluntary movements [42–44]. Subjects with stroke often appear to make jerky and uncoordinated movements, and this partially reflects the fact that they have difficulty controlling for interaction torques during reaching movements [18, 32, 45].

Corrective responses must also deal with the mechanical properties of the limb and display tightly coordinated timing of individual joint kinematics [3]. This requires the integration of proprioceptive information from both the shoulder and elbow to produce the appropriate motor commands to oppose a limb disturbance [9]. Primary motor cortex has been implicated as crucial to this integration of limb feedback for these corrective responses [13]. Therefore, impairments in corrective responses could relate to stroke-related damage of these cortical areas or associated ascending or descending pathways. Subjects with stroke can display abnormal coupling between muscle groups when responding to a perturbation [22]. This is why we also included a joint-based parameter in the present study to quantify specific impairments in coordinating motion at the shoulder and elbow during motor corrections.

The single-joint loads used in the present study required selective increases in motor commands at only one of the two joints. Several subjects (notably left-affected subjects) were identified as impaired in the timing of joint motion during the earliest phase of the corrective response, and in some cases, the impairment was associated with loads at only one of the two joints. However, some subjects with stroke had task impairments in the absence of impairments associated with multijoint coordination. This suggests multiple mechanisms contributing to post-stroke impairments in corrective responses. Future studies are necessary to identify whether subjects with impaired coordination of the two joints during corrective movements also demonstrate impaired coordination during voluntary tasks such as reaching.

### Bilateral impairments in corrective motor responses

A common impact of stroke is impairment in the contralesional limb, but several studies now highlight that impairments can be present in the ipsilesional limb [46–48]. For example, subjects with stroke often display ipsilesional impairments in sensorimotor tasks such as visually-guided reaching [49–55]. In fact, impairments in reaching performance were shown to persist in subjects with subacute stroke even when their ipsilesional clinical scores were normal [56]. Ipsilesional impairments have also been found in online movement adjustments in response to visual perturbations during reaching [57, 58]. As well, short latency responses to a perturbation applied to the ipsilesional arm are attenuated compared to controls [59].

Previous work has shown that long latency responses of the ipsilesional arm can be reduced compared to healthy controls [14] and may not modulate with different types of perturbations [10]. We also found a large proportion of subjects had task-related impairments with their ‘unaffected’ arm (26-63% depending on the parameter). Task-related impairments in the ‘unaffected’ arm were even observed for subjects with CMSA = 7 for their ‘unaffected’ arm.

Although ipsilesional deficits in sensory and motor function can exist, such impairments are usually less than those on the contralesional side [60]. Interestingly, we found that impairments in our postural perturbation task were often qualitatively similar for the ‘affected’ and ‘unaffected’ arms. Taken together, these results suggest that impairments in corrective responses following stroke are more commonly bilateral than other impairments of voluntary control.

The presence of bilateral deficits in our postural perturbation task seems surprising given that these responses are assumed to be generated by the spinal cord and a relatively simple transcortical circuit involving primary somatosensory cortex, cerebellum and primary motor cortex [12, 61–63]. Thus, possible explanations for bilateral deficits include damage to the corticospinal tract fibers that project ipsilaterally to the spinal cord [49, 64], or disinhibition of contralateral primary motor cortex: i.e., diaschisis [65–68]. Deficits in multiple aspects of cognitive and perceptual-motor function may also contribute to impairments in generating feedback responses in either or both limbs. It has been shown that apraxia or deficits in visuo-spatial perception are associated with ipsilesional deficits in hand dexterity [69]. Cognitive impairment such as a deficit in attention has global effects that could also lead to bilateral impairments [70–72].

Another possibility is that bilateral impairments may reflect hemispheric specialization such that damage to one hemisphere would impair particular features of motor control in both limbs [46]. Studies of reaching movements post-stroke reveal left-affected subjects had impairments in endpoint accuracy, whereas right affected subjects had impairments in movement trajectory when using either their ipsilesional [53] or contralesional arm [73]. A study requiring online movement adjustments in response to a visual perturbation during reaching found that left-affected subjects displayed delays in initiating corrective responses and both left- and right- affected subjects displayed large final position errors when using their ipsilesional arm [58]. Endpoint errors were attributed to intersegmental coordination deficits only in right-affected subjects. This supports the idea of the left hemisphere specializing in accounting for intersegmental dynamics and the right hemisphere specializing in controlling position and velocity [47].

Sensory and motor impairments of subjects with stroke tend to be more prevalent in left-affected subjects. Several studies highlight that left-affected subjects tend to more often have impairments in limb motor [50, 54, 55, 74] and proprioceptive function [38, 75]. We also found subjects who were left-affected had impairments in the unaffected limb more often than right affected subjects. This suggests, in general, a greater role of the right as compared to the left hemisphere for sensory and motor processing [76–78].

Finally, online corrective responses to maintain postural control may require other cortical regions beyond primary somatosensory and primary motor cortices [2]. Recent studies highlight the sophistication of corrective responses of non-disabled humans and that such responses begin during the long-latency time period [1, 79]. It has been proposed that rapid corrective responses reflect ongoing voluntary control processes, and thus, implicates much broader cortical circuits beyond a simple transcortical pathway [2]. For example, premotor cortex, primary motor cortex, and cerebellum are active for contra-, but also to a certain degree for ipsilateral limb movements [80–83]. Thus, both motor regions may participate in corrective responses of either limb. Therefore a stroke in either of these regions may lead to deficits in both limbs.

Of particular interest is that about half the subjects with impairments were impaired unilaterally and half were impaired bilaterally. Impairments in controlling the ipsilesional arm tended to be similar in magnitude to impairments in controlling the contralesional arm. The presence of distinct patterns of impairments (bilateral and unilateral) may reflect anatomical differences in lesion size and location for each of sub-group, a focus for future studies.

We found significant correlations between corrective responses with the ‘unaffected’ arm and clinical measures of spatial attention, functional independence, voluntary upper limb control, and strength. This suggests ipsilesional corrective impairments are proportional to overall impairments that impact the ability to function in daily life. It may also relate to the allowed use of the less affected (ipsilesional) arm in many of these clinical assessment measures, such that the test is measuring the ability to compensate with the less affected arm rather than the functional ability of the more affected arm [84]. Future studies can use this posture perturbation task to investigate whether unilateral or bilateral corrective impairments predict differences in recovery trajectories, long-term outcomes, and responsiveness to different rehabilitation therapies.

### Corrective impairments remain with visual feedback

Limb afferent feedback through cortex can occur in ~50-60 ms [2, 12]. Visual feedback is slower, but can influence motor output after ~100 ms when visual disturbances are applied [85–87]. As deceleration times associated with countering the applied load were often half a second or more, it seems surprising that visual feedback was not exploited to initiate the motor response. In fact, except for endpoint errors, we found that subjects’ performance did not improve when visual feedback was provided during the corrective response. This may relate to the fact that subjects with stroke cannot always correct for post-stroke impairments in limb position sense when provided with visual feedback [88].

Interestingly, 74% of subjects with stroke had larger endpoint errors than controls with visual feedback. These findings could not be explained by strength impairments as some subjects who scored poorly in strength returned accurately and some subjects who scored perfect or near perfect in strength had large endpoint errors (Figure 5c,d). As well, subjects’ unaffected arm did not commonly show strength deficits, but were often also impaired. We also found that greater endpoint errors with visual feedback were correlated with delayed return times. Thus, subjects may have difficulties completing a movement back to the target before the end of the trial. These greater endpoint errors with visual feedback could also reflect attentional problems [70–72], as all 5 subjects with stroke whose BIT score was <130 were impaired in this way.

### Limitations

The purpose of the present study was to develop a simple goal-directed task to quantify the ability of subjects to make rapid corrective responses with the upper limb. One limitation of this study is that we did not collect muscle activity to directly measure muscle stretch responses. Subjects were instructed not to co-contract, but some subjects may have had increased muscle activity at perturbation onset. Furthermore, some subjects with stroke may have shown increased resistance to the imposed load due to altered joint stiffness and/or muscle co-activity. As muscle activity was not collected, the direct relationship between altered long latency muscle activity and behavioral responses to a perturbation could not be quantified. We expect that long-latency and potentially later voluntary responses would be reduced in subjects with stroke who had delayed deceleration times in our task. Additionally, subjects with stroke who had bilateral impairments in our task likely have bilateral attenuated long-latency responses, as has been observed previously [14].

A second limitation is the range in time between stroke onset and our clinical and robotic assessments (2 to 50 days). This implies that our sample of subjects with stroke were at different points in their neurological recovery at the time of the assessment. This limitation reflects the inherent variability in the time patients with stroke are admitted for neurorehabilitation at the three facilities in which the study was conducted. One of these facilities moved patients quickly from acute care to rehabilitation while another often did not admit patients to rehabilitation until several weeks post-stroke. As most impairments show some recovery 50 days post-stroke, the incidence of impairments in feedback control of the upper limb may be greater than identified in the present study if subjects were all assessed close to the onset of stroke [89]. We are currently conducting a longitudinal study in which we assess subjects at different time points post-stroke to quantify recovery of feedback control. Nevertheless, even with a cross-sectional design and a range of time post-stroke, the current study still identifies characteristic behavioural impairments in individual subjects with stroke in the use of limb afferent feedback for motor action.

Finally, the definition of impairment based on the 95% performance of healthy controls will create false positives and false negatives. This cutoff makes the task susceptible to incorrectly identifying a subject as impaired (as 5% of control subjects are identified as impaired according to the definition), but was selected to balance sensitivity and specificity.

## Conclusions

The present study demonstrates that stroke can alter corrective actions of both contralesional and ipsilesional arms and this may have important implications for functional abilities. When both upper limbs are impaired, every interaction with an unpredictable external environment carries the risk of spilled drinks, objects dropped and failure in other daily tasks. A risk factor for falling post-stroke is the inability to respond to disturbances in the environment such as a perturbation [90]. Appropriate corrective responses of both the upper [91–93] and lower limbs [94–96] are important to reduce the risk and associated injury of falls [97, 98]. The presence of bilateral impairments in corrective responses may impact the person’s ability to use either arm to catch and stabilize oneself. Furthermore, this could reflect a general difficulty in responding to disturbances with both the upper and lower limbs while walking and standing. Task performance with the ‘unaffected’ arm in the present study correlated with clinical measures of impairment, attention, and functional ability. This may reflect the impact of a person being unable to successfully compensate with the ‘unaffected’ side in these clinical assessments (as well as in activities of daily living). Rehabilitation strategies may need to enlarge their focus to include the ‘unaffected’ side for these bilaterally impaired subjects. This enlarged focus would emphasize the importance of bimanual rehabilitation strategies to rehabilitate both arms, rather than focusing on using the less affected arm to help the performance of the more affected arm.
